# Effects of forage type on the rumen microbiota, growth performance, carcass traits, and meat quality in fattening goats

**DOI:** 10.3389/fvets.2023.1147685

**Published:** 2023-04-27

**Authors:** Zhou-lin Wu, Xue Yang, Jiamin Zhang, Wei Wang, Dayu Liu, Bo Hou, Ting Bai, Rui Zhang, Yin Zhang, Hanyang Liu, Hongwen Hu, Yunhong Xia

**Affiliations:** ^1^Meat Processing Key Laboratory of Sichuan Province, College of Food and Biological Engineering, Chengdu University, Chengdu, China; ^2^Chengdu Academy of Agricultural and Forestry Sciences, Chengdu, China; ^3^Neijiang Academy of Agricultural Sciences, Neijiang, China

**Keywords:** fattening goat, forage treatment, meat quality, rumen microbiota, 16S rRNA

## Abstract

Forages fed to goats influence ruminal microbiota, and further contribute to affect growth performance, meat quality and its nutritional composition. Our objective for current study was to investigate the effects of different forages on growth performance, carcass traits, meat nutritional composition, rumen microflora, and the relationships between key bacteria and amino acids and fatty acids in the *longissimus dorsi* and *semimembranosus* muscles of goats. Boer crossbred goats were separately fed commercial concentrate diet supplemented with Hemarthria altissima (HA), Pennisetum sinese (PS), or forage maize (FG), and then slaughtered 90 days after the beginning of the experiment. Growth performances did not vary but carcass traits of dressing percentage, semi-eviscerated slaughter percentage, and eviscerated slaughter percentage displayed significant difference with the treatment studied. Meats from goats fed forage maize, especially *semimembranosus* muscles are rich in essential amino acids, as well as an increase in the amount of beneficial fatty acids. Our 16S rRNA gene sequencing results showed that the Firmicutes, Bacteroidetes, and Proteobacteria were the most dominant phyla in all groups but different in relative abundance. Further, the taxonomic analysis and linear discriminant analysis effect size (LEfSe) identified the specific taxa that were differentially represented among three forage treatments. The spearman’s correlation analysis showed that rumen microbiota was significantly associated with the goat meat nutritional composition, and more significant positive correlations were identified in *semimembranosus* muscles when compared with *longissimus dorsi* muscles. More specifically, the lipid metabolism-related bacteria Rikenellaceae_RC9_gut_group showed positively correlated with meat amino acid profile, while genera Oscillospiraceae_UCG-005 were positively correlated with fatty acid composition. These bacteria genera might have the potential to improve nutritional value and meat quality. Collectively, our results showed that different forages alter the carcass traits, meat nutritional composition, and rumen microflora in fattening goats, and forage maize induced an improvement in its nutritional value.

## 1. Introduction

Mutton has conventionally been regarded as healthy food and is beneficial to the elderly, children, and pregnant persons (1). In general, goat meat has desirable fatty acids with moderately higher proportion of polyunsaturated fatty acids, lower cholesterol and saturated fat contents compared to beef and pork (2, 3). Meat quality and nutrients are determined by a considerable number of factors whereas genetics, diet, and management are highly ranked factors (4). Diet has a large economic impact on the raising processes as the feeding cost reaches 80% of total cost of production (5). It is important to note that adequate diet is critical for improving the quality and acceptability of animal-derived foods (6).

Forages are regarded as the cheapest and major source of nutrition for ruminant livestock. Ruminants are endowed with the ability to degrade and utilize forages with the help of rumen microbes, while providing adequate energy and protein for the body (7, 8). The rumen of ruminants is an extremely complex microbial ecosystem and hosts 100 trillion (10^14^) microorganisms including bacteria, protozoa, fungi, archaea, and a small proportion of phages. Because these microorganisms are directly involved in the degradation and metabolization of plant materials in the rumen, any changes in nutrient availability will affect rumen microbiota community structure and microbial fermentation patterns (9). Normally, various groups of bacteria have been shown to be associated with the utilization of specific feedstuffs, such as starch or cellulose, which are digested by saccharolytic and cellulolytic bacteria, respectively (10). A forage-based diet is dominated by cellulolytic and fibrolytic bacteria, which degrades the cellulose and hemicellulose, while a concentrate-based diet is dominated by starch-degrading amylolytic bacteria, which ferment the starch and sugars (11). Meanwhile, the composition of the rumen bacterial community has been proven to be associated with feed efficiency (12) and fatty acid composition of meat (3), and fatty acids are closely linked to human health.

Finishing beef are often fed a forage-based diet in order to improve omega-3 polyunsaturated fatty acids, conjugated linoleic acid, and superior nutritional value (13). Similarly, in goats, mixed orchard hays can increase beneficial fatty acids and amino acids of meat, suggesting that feeding suitable type of forage is an important strategy for producing high-quality meat (14). In support of this, effects of forage-based diet types on growth performance, production quality, and rumen microbiome were widely studied (6, 10, 14, 15). However, it is still remains largely unknown about the link between different type of forages and rumen bacterial community composition. In addition, the relationship between rumen microbiota and fatty acids and amino acids in the *longissimus dorsi* and *semimembranosus* muscles of goats is limited. Therefore, in this study, growth performance, carcass traits, meat nutritional composition, rumen microflora of Boer crossbred goats under different forage treatments were determined. Subsequently, the sequential dynamic changes in rumen bacterial community composition and their relationships with the fatty acids and amino acids were analyzed comprehensively using high-throughput sequencing approach. Our work aimed to compare rumen bacterial community of diets differing in forage type, and reveal the dominant bacteria related to contributing to a good nutritional quality meat of goats.

## 2. Materials and methods

### 2.1. Ethics statement

All experimental procedures involved in this study were conducted in accordance with the guidelines duly approved by Institutional Animal Care and Use Committee of Chengdu University (SSXY-600008).

### 2.2. Animal treatments and sample collection

In this study, a total of 15 healthy 6-month-old male Boer crossbred goats with an average body weights (BW) of 19.61 ± 3.25 kg were enrolled and housed in individual wooden pens (1.20 m length × 0.80 m width × 1.50 m height) on a raised slatted floor. Goats were randomly divided into three forage treatment groups of concentrate + forage, within each group one of the forages including Hemarthria compressa, Pennisetum sinese, or forage maize was used, and then designated as HA, PS, and FG group, respectively. For each group, the concentrate was offered 3.5 kg per day for each, whereas forage and water were both offered *ad libitum*. The chemical compositions of the concentrate and forages are shown in Supplementary Table S1.

The study commenced with a 7-day adaptation period and lasted for 90 days. Of these enrolled goats, two animals in the HA group were sick and treated with drug during the experiment, these two goats were removed from the herd. Finally, a subset of 13 goats were obtained and then divided into three groups of HA (*N* = 3), PS (*N* = 5), and FG (*N* = 5), and all the animals were weighed and slaughtered according to standard protocol at the end of the experiment. Animals were subjected to electrical stunning at 220 voltages followed by exsanguination, skinning, evisceration, and washing procedures. Immediately after slaughter, the rumen fluid samples were collected from each goat, and the rumen contents were removed by using three layers of cheesecloth. Subsequently, the liquid fractions were transferred into plastic bottles, and stored at −80°C until further evaluation. On the other hand, the carcasses were cooled to 4°C for 24 h followed by further analyses.

### 2.3. Meat quality measurements

The *longissimus dorsi* and *semimembranosus* muscles were cut to measure the meat quality traits according to previous reports (3, 16). Briefly, the pH values were measured at 24 h (pH_24h_) after slaughter. Meat color measurements cover indicators L* (lightness), a* (redness), and b* (yellowness) after slaughter 24 h (L_24h_, a_24h_, and b_24h_). Drip loss, cooking loss, crude protein, and ash content were determined as described previously (3, 17). Amino acid profile and fatty acid composition were determined using gas chromatography–mass spectrometry (GC–MS 7890B-5977A, Agilent, Palo Alto, CA, United States) and liquid chromatography–mass spectrometry (Liquid phase was performed on Thermo Ultimate 3,000 system, Thermo Fisher Scientific Inc., Waltham, MA, United States; Mass Spectrometry was performed on Thermo Q Exactive Focus mass spectrometer, Thermo Fisher Scientific Inc., Waltham, MA, United States), respectively.

### 2.4. DNA extraction and sequencing

Frozen rumen liquid (approximately 15 g) from each animal was subjected to microbial genomic DNA isolation using QIAamp DNA Stool Mini Kit (Qiagen, Shanghai, China) according to the manufacturer’s protocol. The DNA concentration and purity were evaluated using a NanoDrop ND-1000 spectrophotometer (NanoDrop Technologies, Montchanin, DE, United States) and gel electrophoresis, respectively. Amplicon libraries targeting the V3–V4 hypervariable region of the bacterial 16S rRNA gene were amplified by PCR using primers with barcoded tags (338F, 5′-ACTCCTACGGGAGGCAGCA-3′, 806R: 5′-GGACTACHVGGGTWTCTAAT-3′). The reaction was performed with the following cycle parameters: initial denaturation for 3 min at 95°C, 30 cycles of 95°C for 30 s, 55°C for 30s, and 72°C for 30s, and a final extension at 72°C for 10 min. The PCR products were separated on 1.5% agarose gel electrophoresis. Qualified amplicons were used to produce sequencing libraries using Illumina TruSeq (Illumina, San Diego, CA, United States) following manufacturer’s specifications. Finally, the libraries were diluted and mixed in proportion and sequenced on Illumina HiSeq 2,500 platform for generating 250 bp paired-end reads.

### 2.5. Bioinformatics and data analysis

Paired-end reads were assigned to samples based on their unique barcode, and the high-quality clean reads were obtained by using the Cutadapt software (1.9.1) (18). Sequences analysis was performed by Qiime2 software (19). By which, representative sequences were established as operational taxonomic units (OTUs) and aligned through DEBLUR program (20) integrated within QIIME2. Then, OTUs were taxonomically classified and grouped by comparison with those in the Silva reference sequences (138 clustered at 99% similarity). Successive analyses of alpha diversity and beta diversity were conducted. The alpha diversity metrics including community richness parameters (Chao1 and Observed features) and diversity parameters (Shannon and Simpson indices) were calculated and significant differences between groups were assessed with the Kruskal-Wallis test (19, 21). The beta diversity metrics including Bray Curtis, Jaccard, Weighted UniFrac, and Unweighted UniFrac metric were calculated and significant differences were assessed using a PERMANOVA analysis (19, 21). Moreover, the rarefaction and rank curves were generated to assess the sequencing depth, richness, and evenness. To identify the bacterial taxa that were differentially represented at the genus or higher taxonomic levels, linear discriminant analysis coupled with effect size (LEfSe) was performed (22), where linear discriminant analysis (LDA) method was used to rank the features differing between the groups, and a LDA score > 2 was considered significant. Statistical analyses were performed using R (v4.1.3) software.^1^ The criterion of significance was conducted at *p* < 0.05 and the values were presented as the means. Spearman’s correlation between the identified ruminal genera and the contents of amino acids and fatty acids of all the enrolled goats was performed using corrplot package the R language (v4.1.3), and *p*-values <0.05 were selected as statistically significant.

### 2.6. Statistical analysis

Data were analyzed based on a general linear model of analysis of variance (ANOVA) using SPSS 21.0 (IBM Corp., New York, United States). Differences between mean values of different forage treatments were obtained by Fisher’s LSD multiple range test, and a statistically significant difference was defined at *p* < 0.05.

## 3. Results

### 3.1. Effects of forage treatment on the growth performances and carcass characteristics

There was no significant difference in the initial body weight, the final body weight, and average daily weight gain of the goats among the forage treatment groups (*p* > 0.05; Table 1). Further, we observed no effect of forage treatment on the carcass parameters of semi-eviscerated weight and eviscerated weight. However, animals from HA group had significantly (*p* < 0.05) highest semi-eviscerated slaughter percentage and eviscerated slaughter percentage as well as the higher dressing percentage as compared to PS and/or FG group (Table 1).

**Table 1:** Growth and carcass traits in goats under different forage treatments.

Traits^1^		Group			*p* value	
		HA	PS	FG	Group	Tissue
Initial body weight (kg)		21.42 ± 3.75	19.49 ± 4.23	19.55 ± 2.65	0.478	/
Final body weight (kg)		33.47 ± 5.71	30.97 ± 5.68	32.84 ± 2.26	0.477	/
ADG^1^ (kg/d)		0.13 ± 0.02	0.12 ± 0.09	0.14 ± 0.03	0.635	/
Dressing percentage (%)		52.41 ± 1.14^a^	50.48 ± 2.04^ab^	49.17 ± 0.32^b^	0.011	/
Semi-eviscerated weight (kg)		24.74 ± 4.12	21.93 ± 4.19	22.49 ± 1.75	0.286	/
Semi-eviscerated slaughter percentage (%)		73.94 ± 0.53^Aa^	70.72 ± 2.02^ABb^	68.84 ± 1.97^Bb^	0.002	/
Eviscerated weight (kg)		18.30 ± 3.49	16.34 ± 3.34	16.89 ± 1.17	0.345	/
Eviscerated slaughter percentage (%)		54.55 ± 1.18^A^	52.60 ± 1.89^AB^	51.42 ± 0.29^B^	0.009	/
*Longissimus dorsi*	Drip loss (%)	14.47 ± 2.07^a^	9.92 ± 2.29^b^	11.03 ± 2.03^ab^	0.016	0.113
	Cooking loss (%)	45.34 ± 2.79	45.67 ± 2.65	45.70 ± 0.92	0.825	0.022
	Moisture content (%)	75.49 ± 0.77	75.90 ± 1.07	75.30 ± 1.16	0.396	0.003
	Crude protein (%)	82.82 ± 3.35	79.16 ± 7.24	74.21 ± 8.39	0.131	0.354
	Fatty acid (%)	6.12 ± 1.26	10.00 ± 6.48	14.60 ± 7.15	0.087	0.133
	Ash (%)	7.91 ± 0.32^a^	4.70 ± 1.69^b^	6.26 ± 2.29^ab^	0.035	0.468
	H_24h_	5.44 ± 0.08	5.50 ± 0.12	5.52 ± 0.17	0.409	<0.001
	L_24h_	43.77 ± 1.29^Bb^	45.25 ± 2.35^ABb^	48.05 ± 0.97^Aa^	0.006	0.257
	a_24h_	16.77 ± 1.43	17.28 ± 2.50	16.66 ± 2.31	0.671	0.048
	b_24h_	7.73 ± 1.65	7.03 ± 0.73	8.27 ± 1.02	0.100	0.529
*Semimembranosus*	Drip loss (%)	12.30 ± 1.99	13.51 ± 1.79	12.84 ± 2.89	0.493	0.113
	Cooking loss (%)	42.47 ± 1.41	44.13 ± 2.58	43.06 ± 3.57	0.445	0.022
	Moisture content (%)	76.32 ± 0.73	76.83 ± 0.96	77.05 ± 0.99	0.309	0.003
	Crude protein (%)	83.09 ± 1.61^a^	80.54 ± 2.41^ab^	78.22 ± 3.88^b^	0.049	0.354
	Fatty acid (%)	7.21 ± 1.67	7.68 ± 0.79	8.77 ± 1.29	0.109	0.133
	Ash (%)	7.85 ± 0.41^A^	4.11 ± 0.33^B^	8.49 ± 0.61^A^	<0.001	0.468
	pH_24h_	5.84 ± 0.15^a^	5.61 ± 0.10^b^	5.72 ± 0.07^ab^	0.012	<0.001
	L_24h_	42.82 ± 2.87	45.26 ± 2.07	45.71 ± 2.51	0.134	0.257
	a_24h_	20.72 ± 1.97	19.59 ± 3.21	17.58 ± 3.38	0.193	0.048
	b_24h_	8.67 ± 3.65	8.48 ± 1.17	7.35 ± 2.00	0.428	0.529

Values within a row with different lowerscripts and/or superscripts differ significantly at *p* < 0.05 and *p* < 0.01, respectively. ^1^dage, average daily weight gain, pH_24h_ = pH of muscles after slaughter 24 h, L_24h_, a_24h_, b_24h_ = Lightness, redness and yellowness of muscles after slaughter 24 h.

Most of the carcass traits between *longissimus dorsi* and *semimembranosus* muscles were similar, except for cooking loss, moisture content, pH_24h_, and a_24h_. For these characteristics, the *longissimus dorsi* muscles presented significant higher cooking loss (*p* < 0.05), pH_24h_ (*p* < 0.01) and a_24h_ (*p* < 0.05), and lower moisture content (p < 0.01) when compared with *semimembranosus* muscle (Table 1). As for forage treatments, there was significant difference in the drip loss (*p* < 0.05), ash (*p* < 0.05), and L_24h_ (*p* < 0.01) of *longissimus dorsi* muscles among the three groups. In addition, animals from HA group presented significant higher crude protein, ash, and pH_24h_ of *semimembranosus* muscle, compared with PS and/or FG group (Table 1).

### 3.2. Effects of forage treatments on the amino acid and fatty acid composition

A total of 17 amino acids and 37 fatty acids were tested to examine whose relative content, and most of them did not differ among the forage treatment groups (Supplementary Table S2). The contents of serine (Ser) and proline (Pro) were significantly changed in both types of muscles. However, no fatty acids in *longissimus dorsi* muscles were found to be affected by the forage treatments. In *semimembranosus* muscles, a total of nine amino acids and three fatty acids were affected by forage treatments. Interestingly, the content of all these three fatty acids of linoleic acid (C18: 2n6C), arachidonic acid (C20: 4n6), and docosahexaenoic acid (C22: 6n3) were significant higher for FG than PS groups (*p* < 0.05; Table 2).

**Table 2:** Significantly changed amino acids (g/100 g dry matter) and fatty acids (g/100 g dry matter) of goat meat under different forage treatments.

Traits		Group		
		HA	PS	FG
*Longissimus dorsi*	Ser	2.32 ± 0.11^ABa^	2.56 ± 0.40^Aa^	1.83 ± 0.14^Bb^
	Pro	1.73 ± 0.74^ab^	3.12 ± 2.13^a^	0.91 ± 0.76^b^
*Semimembranosus*	Asn	6.20 ± 0.24^A^	3.86 ± 0.11^B^	4.47 ± 0.82^B^
	Glu	11.77 ± 0.30^a^	10.62 ± 0.20 ^b^	11.33 ± 0.87^b^
	Ser	2.25 ± 0.09^ABa^	2.64 ± 0.06^Aa^	1.72 ± 0.37^Bb^
	Thr	2.90 ± 0.10^A^	2.97 ± 0.03^A^	2.52 ± 0.34^B^
	Ala	4.29 ± 0.16^a^	3.84 ± 0.14^b^	4.11 ± 0.26^ab^
	Cys	0.44 ± 0.04^B^	0.50 ± 0.02^A^	0.40 ± 0.02^B^
	Val	4.92 ± 0.17^A^	4.22 ± 0.03^B^	4.78 ± 0.37^A^
	Phe	6.33 ± 0.26^A^	5.36 ± 0.06^B^	6.14 ± 0.51^A^
	Pro	1.83 ± 0.62^b^	4.02 ± 1.15^ab^	4.72 ± 2.05^a^
	C18:2n6C (linoleic acid)	0.76 ± 0.35^ab^	0.51 ± 0.20^b^	0.97 ± 0.38^a^
	C20:4n6 (arachidonic acid)	0.30 ± 0.19^ab^	0.22 ± 0.12^b^	0.47 ± 0.12^a^
	C22:6n3 (docosahexaenoic acid)	0.06 ± 0.03^ab^	0.04 ± 0.03^b^	0.08 ± 0.02^a^

The results are presented as means and standard errors. Values within a row with different lowerscripts and superscripts differ significantly at *p* < 0.05 and *p* < 0.01, respectively.

### 3.3. Rumen bacterial community structure

**Figure 1:**
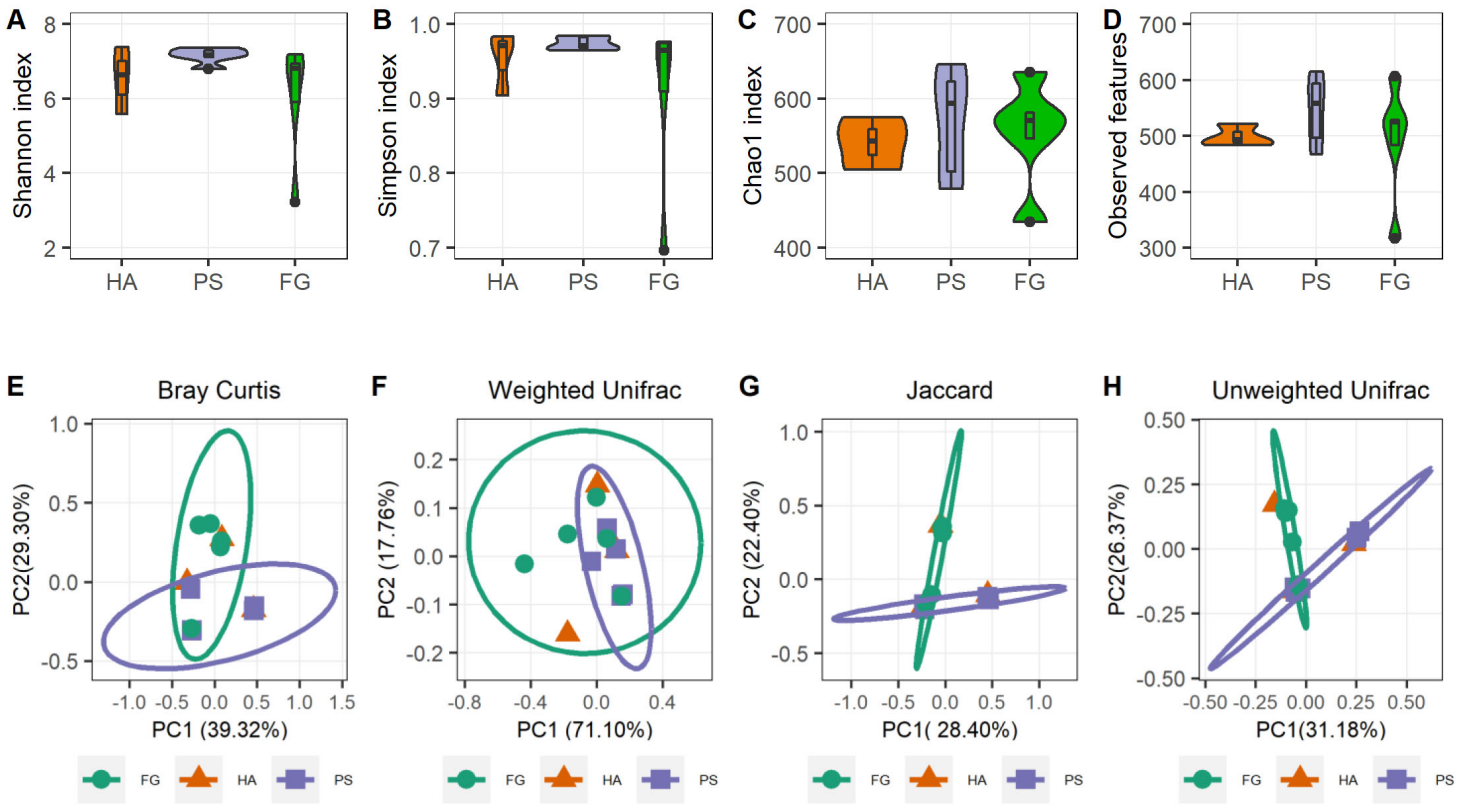
Rumen microbiota diversities of goats grouped by different forage treatments. Comparison of the diversity (Shannon and Simpson indices) **(A,B)** and richness (Chao1 and Observed features) **(C,D)** of the rumen microbiota community. The overall rumen microbiota structures showed by principal coordinate analysis (PCoA) of Bray Curtis distances **(E)**, Weighted UniFrac distances **(F)**, Jaccard distances **(G)**, and Unweighted UniFrac distances **(H)**.

A total of 1,039,145 high-quality reads remained after quality control processing and eliminating the unqualified data, with an average of 79,934 paired-end reads per sample. All sequences were subjected to OUT picking according to DEBLUR program, and herein produced a total of 1,489 OTUs, and these OTUs were assigned into 10 phyla, 13 classes, 37 orders, 66 families, and 119 genera. Alpha diversity consists of community diversity (Shannon and Simpson indices) and richness (Chao1 and Observed features) were assessed and compared for each treatment. Although variation in the inter-animal dynamics of the alpha diversity was observed, no significant differences in the overall alpha diversity indices of the rumen microbiome were found (*p* > 0.05) (Figures 1A–D). These results indicated that the forage treatments did not significantly change the rumen microbial abundance and diversity of goats. We next assessed the dissimilarities in community structure and membership of rumen microbiome between groups, and there were no significant difference in beta diversity indices (Supplementary Figure S1), the PCoA plots based on beta diversity metrics (Bray Curtis, Weighted UniFrac, Jaccard, and Unweighted UniFrac metric) were shown in Figures 1E–H. Beside HA group that has only three samples, individuals in the PS and FG group were clustered separately, indicating bacterial communities were positively correlated with the treatment of forage types.

### 3.4. Composition analysis of the rumen microbiota

**Figure 2:**
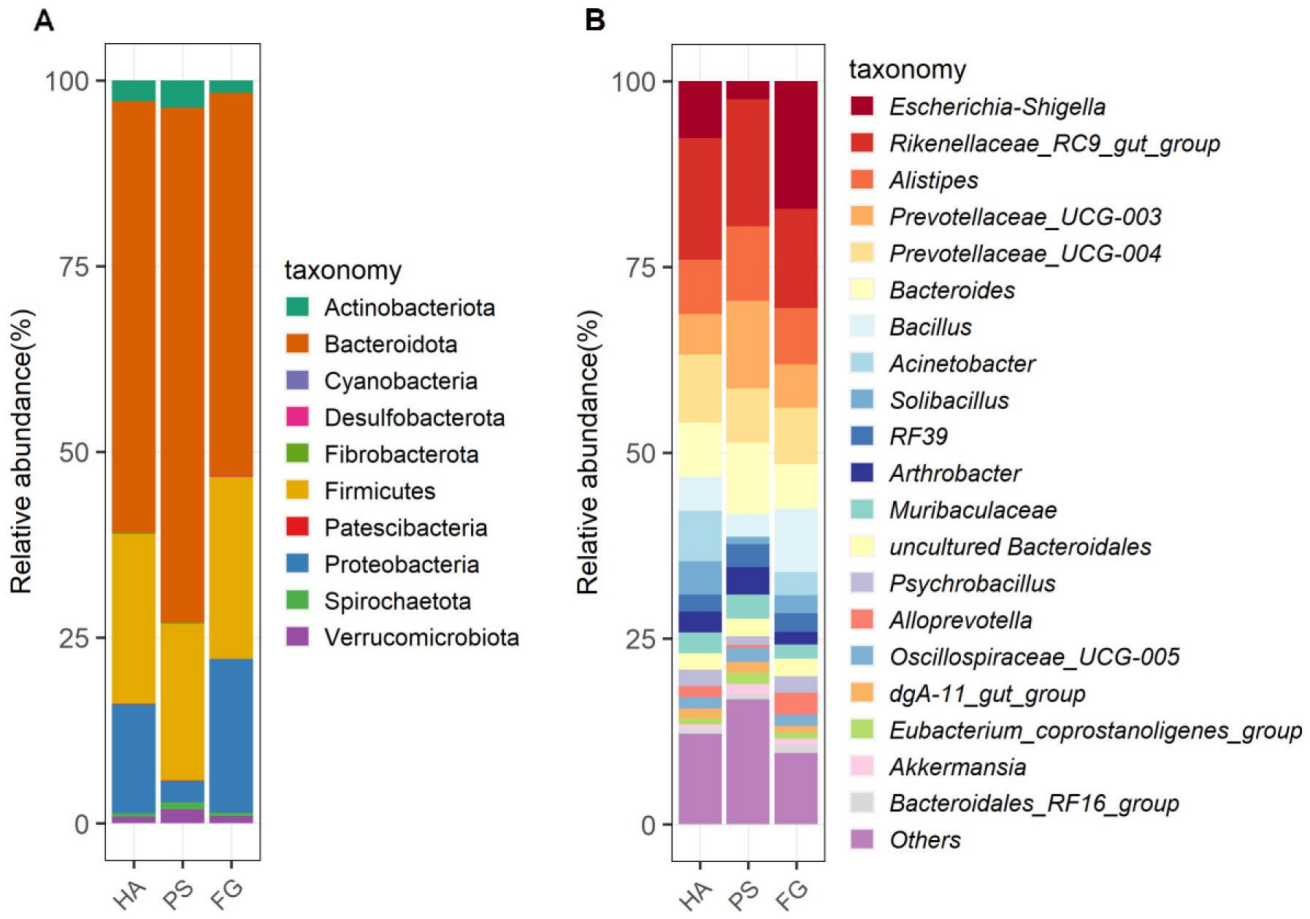
The composition and relative abundance of rumen microbial community of goats. Microbial community bar plot of phyla in rumen **(A)**. Microbial community bar plot of top 20 genera in rumen **(B)**.

Among these taxonomically OTUs, Bacteroidota and Firmicutes were absolutely predominated phyla in all of the three groups of goats, followed by Proteobacteria (Figure 2A). These three phyla accounted for 95.72, 93.28, and 96.70% of the sequences for HA, PS, and FG group, respectively. At the genus level, the relative abundance of the top 20 genera together made up 87–90% of the total composition, and Rikenellaceae_RC9_gut_group were the dominant genera in HA and PS group, reaching a proportion of 16.42 and 17.07%, respectively. Whereas Rikenellaceae_RC9_gut_group in the FG group were found to be the subdominant genera with an abundance of 13.37%, following *Escherichia-Shigella* (17.20%). In addition, Prevotellaceae_UCG-004 and Prevotellaceae_UCG-003 were subdominant genera in HA and PS group, accounting for 9.15 and 11.81%, respectively (Figure 2B).

**Figure 3:**
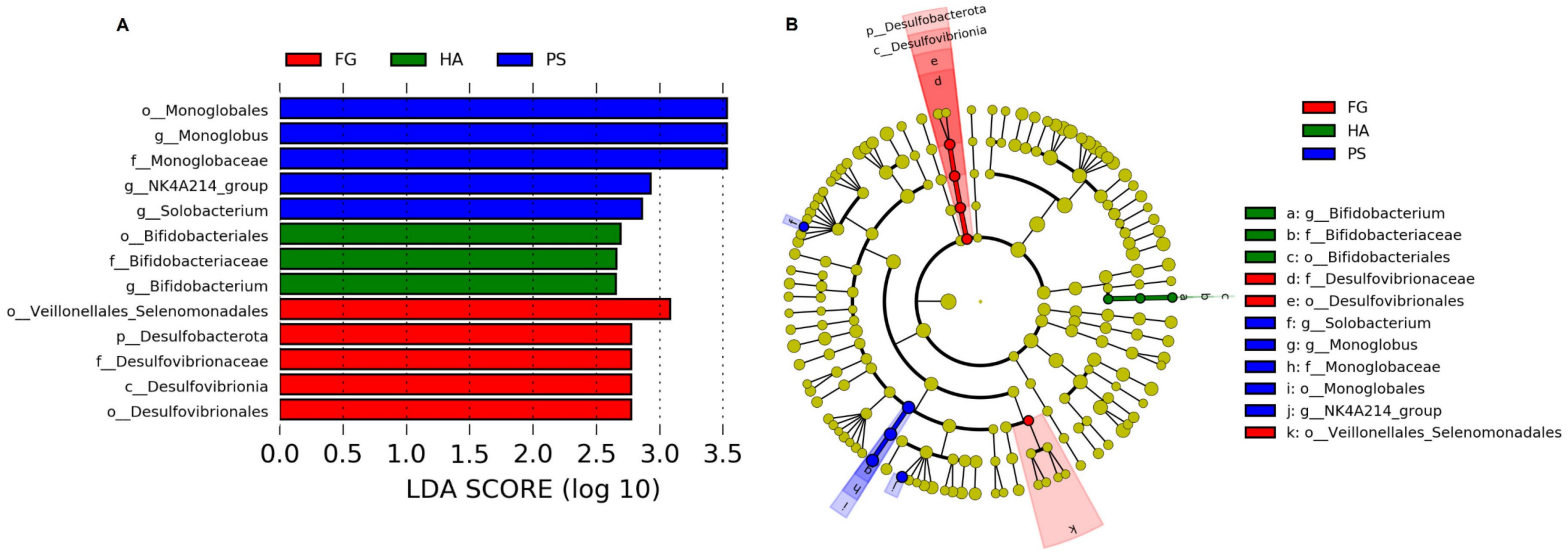
Linear discriminant analysis effect size (LEfSe) analysis integrated with linear discriminant analysis (LDA) revealed differentially abundant phylotypes in different groups. LDA scores indicated differences in abundance among forage treatments (LDA scores >2.0) **(A)**. Cladogram obtained from LEfSe analysis revealed the different taxa in microbiota of different groups of goats **(B)**.

We further detected the specific bacteria associated with dietary treatment using Linear discriminant analysis Effect Size (LEfSe) analysis. As shown in Figure 3, a total of three, five, and five bacterial taxa that were abundant in HA, PS, and FG group, respectively. At the genus level, *Bifidobacterium* was significantly enriched in HA group, while *Monoglobus*, *Selenomonas*, and NK4A214_group were mostly associated with PS group based on LEfSe (Figure 3A). Furthermore, a cladogram representing the taxonomic hierarchical structure of rumen microbiota indicated significant difference phylogenetic distributions among different groups (Figure 3B). These results showed a remarkable difference in rumen microbiota composition due to different dietary treatment.

### 3.5. Correlation analysis of rumen microflora and meat quality composition

**Figure 4:**
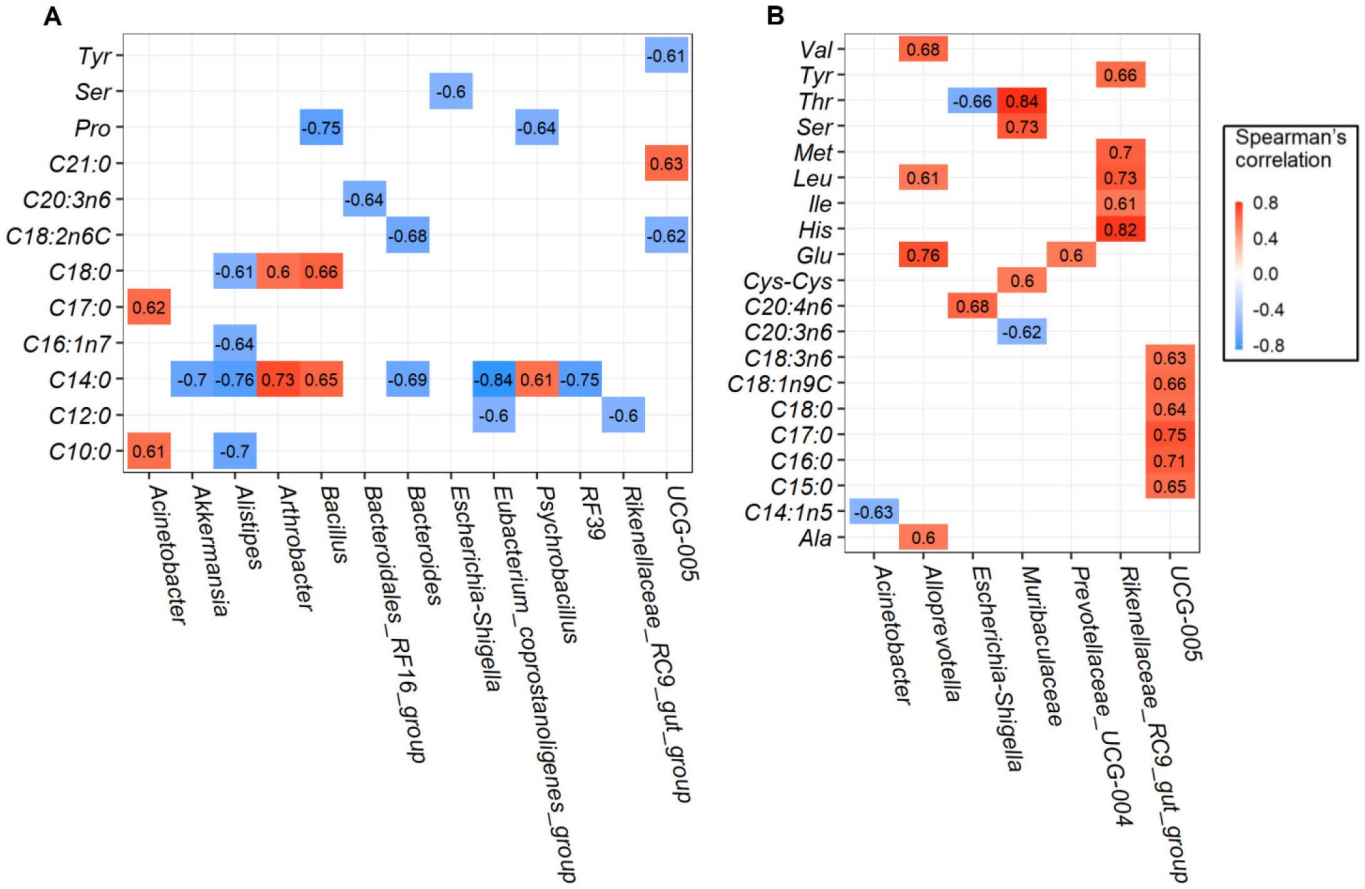
Correlation analysis between the rumen bacteria and amino acids and fatty acids. Significant correlations between the content of top 20 genera and amino acids and fatty acids in *longissimus dorsi* **(A)**, and *semimembranosus* **(B)**.

Robust correlations between amino acids and fatty acids and major rumen bacterial composition at the genus level were conducted, only the spearman correlation coefficients |*r*| > 0.6 and *p*-values <0.05 are drawn in the heat map (Figure 4). For *longissimus dorsi* muscles, bacteria genera of Oscillospiraceae_UCG-005, *Escherichia-Shigella*, *Bacillus* and *Psychrobacillus* was negatively correlated with amino acids of tyrosine (Tyr), serine (Ser), and proline (Pro). Saturated fatty acids such as capric acid (C10: 0), lauric acid (C12: 0), myristic acid (C14: 0), heptadecanoic acid (C17: 0), stearic acid (C18: 0), and heneicosanoic acid (C21: 0) were strongly correlated with several bacteria. Among which, myristic acid (C14: 0) was positively correlated with *Arthrobacter*, *Bacillus*, and *Psychrobacillus* abundances, but negatively correlated with Eubacterium_coprostanoligenes_group, *Alistipes*, *RF39*, *Akkermansia*, and *Bacteroides* abundances (Figure 4A). The effects of rumen bacteria abundances on amino acid and fatty acid content of the *semimembranosus* muscles are given in Figure 4B, Rikenellaceae_RC9_gut_group abundance was found to be positively correlated with amino acid content of tyrosine (Tyr), methionine (Met), leucine (Leu), isoleucine (Ile), and histidine (His). In addition, genera *Alloprevotella* and *Muribaculaceae* were positively correlated with several amino acids. Interestingly, Oscillospiraceae_UCG-005 abundance was positively correlated with fatty acid composition except amino acid composition. Together, we found more significant positive correlations in *semimembranosus* muscles were identified when compared with *longissimus dorsi* muscles.

## 4. Discussion

Roughage is an important part of feed for ruminants. Although a large number of forage species can utilize to provide roughage for domestic animals, there are diverse agronomic requirements for effective production forages (23). Meanwhile, supplementation with local and traditional forage is a strategy for reducing feeding cost and improve profitability for smallholders, and concentrate feeds on growth performance is often unsatisfactory in goat rearing (24). It is after these realizations that forage species namely Hemarthria altissima, Pennisetum sinese, and forage maize were chosen to fed local Boer crossbred goats in this study. Although goat growth rate was not affected by the forage type, animals from the HA group had the highest semi-eviscerated slaughter percentage and eviscerated slaughter percentage with the higher dressing percentage among the three groups (Table 1). This would imply that Hemarthria altissima is more suitable to improve the production efficiency.

Water-holding capacity is defined as the ability of meat to bind water and, therefore, always linked to the sensory properties of meat such as juiciness, texture, and flavor (25). The water released can be described as drip loss, and which is inversely related to water-holding capacity. In this study, the moisture content and cooking loss of goats were similar with no differences among different forage treatments, whereas animals fed with Hemarthria altissima forage have higher drip loss of *longissimus dorsi* muscles as compared to other two types of forages. This may be due to the different organizational structure of the meat between these three forage treatments. Meat color is one of the most important factor that can affect consumers’ initial selection and purchase decision, and which may be contribute to combined effects of breed, aging, diet, intramuscular fat, and meat pH (3, 26). According Realini et al. (27), pasture-feed steers had darker color of *longissimus dorsi* muscles as compared to concentrate-fed steers. In the current study, the *longissimus dorsi* muscles from HA group had significantly lower L_24h_ values. The possible explanation was that it was related to factors such as forage composition and physical activities, which requires further investigation.

Amino acids are the basic components of animal protein, and changes in types and concentrations directly affect the nutritional value and flavor of meat. Forage treatments affected the amino acid profile of goat meat, especially whose profiles in *Semimembranosus* muscles. Goats fed forage maize had significant lower content of five of the non-essential amino acids measured (Asn, Glu, Ser, Ala, and Cys) but higher content of Pro than those of the other two groups in *Semimembranosus* muscles. Meanwhile, essential amino acids are critical for the body, and which is usually used to evaluate the biological value of protein (28). In this study, the contents of essential amino acid of threonine (Thr) was lower and other two essential amino acids of valine (Val) and phenylalanine (Phe) were higher in *Semimembranosus* muscles of goats fed forage maize. This result was consistent with the observation that the amino acids profile of goat meat protein was significantly affected by diet (29). Overall, meats from goats fed forage maize, especially *Semimembranosus* muscles are rich in essential amino acids, can be good sources of proteins for humans.

In addition to amino acid profile, a previous publication has showed that diet had effects on other physicochemical properties of goat meat, such as fatty acid profile and sensory qualities (30). In this study, the predominate fatty acids detected in *longissimus dorsi* and *semimembranosus* muscles were palmitic acid (C16: 0), oleic acid (C18: 1n9c), linoleic acid (C18: 2n6c), stearic acid (C18: 00), and arachidonic acid (C20: 4n6) (Supplementary Table S2). Similar results have been reported in a previous study conducted on Korean native black goats (6). The composition and concentration of fatty acids of *longissimus dorsi* muscles were not influenced by the forage treatments. However, goats fed forage maize had a significant higher contents of linoleic acid (C18: 2n6c), arachidonic acid (C20: 4n6), and docosahexaenoic acid (C22: 6n3) in the *semimembranosus* muscles compared with other two treatments (Table 2). Especially, docosahexaenoic acid has long been proposed to bestow health benefits by improving blood pressure control, attenuating the progression of Alzheimer’s disease (31). Therefore, the docosahexaenoic acid is recognized as a beneficial dietary constituent, and goat meat is a desirable candidate for dietary docosahexaenoic acid enrichment (32). This suggests that, goats fed forage maize induced an increase in the amount of beneficial fatty acids and, therefore, improvement of its nutritional value. The fatty acids of meat mainly affected by diet composition and rumen microbiota because which absorbed by the duodenum are mainly from dietary origin as well as the result of rumen microbial biohydrogenation of dietary lipids (33). Moreover, several bacterial species are fond to be associated with beneficial fatty acids, such as *Butyrivibrio_2* was positively correlated to the *α*-linolenic acid (C18: 3 n-3, ALA) and conjugated linoleic acid (CLA) contents in sheep (34). In addition, several studies highlighted that the fat content of meat could be enhanced by providing a high energy diet (35). Here we analyzed the fatty acids of *semimembranosus* muscles among different forage treatments, it should be noted that the fatty acids in each diet of HA, PS, and FG as well as which in rumen remains unexplored. Future research would have to address these questions.

In the present study, neither the alpha diversity nor the relative abundances of main phyla were affected significantly by these three forage treatments. The relative abundances of Bacteroidota, Firmicutes, and Proteobacteria showed to be predominated phyla in the three groups (Figure 2A), which is consistent with the previous results conducted in goats (14) and other ruminants (36). At the genus level, Rikenellaceae_RC9_gut_group remained the dominant species in HA and PS group, whereas which were sub-dominated in FG group. Rikenellaceae_RC9_gut_group belong to the Rikenellaceae family, which play a key role in the digestion of crude fiber, and whose abundance decreased along with the content reduction of neutral detergent fiber in the diet (37). However, the highest neutral detergent fiber content in forage maize was found to be associated with the decrease of Rikenellaceae_RC9_gut_group in the rumen of FG goats. Future research would have to address this question. *Escherichia-Shigella* is a well-known member of the normal intestinal microflora of animals, and which is a potential pathogen known to delay the establishment of the anaerobic rumen environment (38). The high abundance of this genus in FG animals might be related to the ruminal fermentation parameters, ruminal enzymic activities, and ruminal epithelium development (39). However, the relationship between the *Escherichia-Shigella* and rumen fermentation parameters remains poorly understood and deserves further investigation.

Rumen bacteria are closely related to animal production and meat quality traits (40). Accordingly, we assessed whether the correlation existed between the bacterial genera and amino acids and/or fatty acids. The Rikenellaceae_RC9_gut_group genera played vital roles in carbohydrates degradation (41, 42), and was reported to be positively correlated with fatty acids production (14). However, no published research to date has yet to explore the correlation between genera Rikenellaceae_RC9_gut_group and amino acids. Results of the present study showed that strong positive correlations between bacterial genera and amino acids were found in the *semimembranosus* muscles, especially the lipid metabolism-related bacteria Rikenellaceae_RC9_gut_group were found to be positively correlated with amino acid content of tyrosine (Tyr), methionine (Met), leucine (Leu), isoleucine (Ile), and histidine (His), indicating this genus has played important role in modulating meat amino acid in ruminants. In addition, genera Oscillospiraceae_UCG-005 were positively correlated with several fatty acids such as pentadecanoic acid (C15: 0), palmitic acid (C16: 0), heptadecanoic acid (C17: 0), stearic acid (C18: 0), oleic acid (C18: 1n9C), and *γ*-linolenic acid (C18: 3n6), therefore, it is tempting to explore whether Oscillospiraceae_UCG-005 could improve goat meat nutritional value by increasing the amount of beneficial fatty acids.

## 5. Conclusion

Feeding suitable type of local and traditional forage is an important strategy for producing high-quality goat meat as well as reducing feeding cost for smallholders. In this study, we found that forage maize is more suitable to improve the production efficiency, and *longissimus dorsi* muscles of goats fed forage maize had significantly lower L_24h_ values. In addition, goats fed forage maize can increase beneficial fatty acids and amino acids and, thereby induce an improvement in their meat nutritional value. Further, our 16S rRNA gene sequencing results showed that rumen microbiota was significantly associated with the goat meat nutritional compositions, and bacteria Rikenellaceae_RC9_gut_group and Oscillospiraceae_UCG-005 showed significantly positive correlated with the beneficial fatty acids contents.

## Data availability statement

The datasets presented in this study can be found in online repositories. The names of the repository/repositories and accession number(s) can be found in the article/Supplementary material.

## Author contributions

Zl-W, WW, JZ, and YX: conceived and designed the experiments. Zl-W, XY, HL, BH, TB, DL, YZ, and RZ: performed the experiments. Zl-W, XY, and HH: analyzed the data. Zl-W: wrote the paper. Zl-W, XY, and YX: reviewed and edited the manuscript. All authors contributed to the article and approved the submitted version.

## Funding

This work was supported by the Youths Fund of Natural Science Foundation in Sichuan Province (No. 2022NSFSC1746), the National Key Research and Development Program of China (No. 2021YFD1100201), the Key Research and Application Program of Chengdu (No. 2022-YF09-00038-SN), Research on Modern Processing Technology and Quality Improvement Technology of Mutton Ham (No. CC18Z03), and the Special Project for Local Science and Technology Development with China Central Government Guidance (No. 2020ZYD067).

## Conflict of interest

The authors declare that the research was conducted in the absence of any commercial or financial relationships that could be construed as a potential conflict of interest.

## Publisher’s note

All claims expressed in this article are solely those of the authors and do not necessarily represent those of their affiliated organizations, or those of the publisher, the editors and the reviewers. Any product that may be evaluated in this article, or claim that may be made by its manufacturer, is not guaranteed or endorsed by the publisher.

## Footnotes

^1^ https://www.r-project.org/
