# Supplementary material for: Effects of forage type on the rumen microbiota, growth performance, carcass traits, and meat quality in fattening goats
Source: Front Vet Sci. 2023 Apr 27;10:1147685. doi: 10.3389/fvets.2023.1147685 (PMC10172669; doi:10.3389/fvets.2023.1147685)
Supplement: Supplementary file 1 [file Table_1.DOCX]

Supplementary Material

# **Supplementary Tables**

**Table S1:** Ingredient and nutrient composition of the diets.

| **Item** | **Parts** | | |
| --- | --- | --- | --- |
| **concentrate** |  | | |
| **Diet component** | **Percentage composition** | | |
| alfalfa grass | 15.50 | | |
| oat grass | 10.10 | | |
| corn silage | 17.15 | | |
| corn | 22.05 | | |
| wheat bran | 2.60 | | |
| soya bean meal | 8.85 | | |
| rapeseed meal | 1.30 | | |
| cottonseed meal | 1.30 | | |
| whole cottonseed | 11.00 | | |
| expand soybean | 4.40 | | |
| common salt | 0.30 | | |
| calcium hydrophosphate | 1.55 | | |
| microelement additive | 0.50 | | |
| magnesium oxide | 0.30 | | |
| baking soda | 1.80 | | |
| fatty acid calcium | 1.30 | | |
| **Nutrient component** | **Content (% DM basis)** | | |
| RUP | 31.82 | | |
| CP | 17.10 | | |
| NDF | 40.98 | | |
| ADF | 26.43 | | |
| Ca | 0.89 |  |  |
| P | 0.58 |  |  |
| Na | 0.64 |  |  |
| K | 1.34 |  |  |
| Mg | 0.40 |  |  |
| NEL, MJ/kg of DM | 6.10 MJ/Kg |  |  |
|  |  |  |  |
| **FORAGE** | **Hemarthria compressa** | **Pennisetum sinese** | **forage maize** |
| **Nutrient component** | **Content (% DM basis)** |  |  |
| CP | 8.3 | 7.23 | 12.19 |
| ADF | 39 | 44.3 | 30.98 |
| NDF | 56.2 | 70.02 | 70.37 |
| Lingnin | 4.9 | 5 | 5.3 |
| NFC | 21.47 | 15.58 | 18.45 |
| Starch | 3 | 1.8 | 2.2 |
| Fat | 2.5 | 1.93 | 2.46 |
| Ash | 7.22 | 4.88 | 6.35 |
| TDN | 53.3 | 57.67 | 58.45 |
| Ca | 0.25 | 0.33 | 0.8 |
| P | 0.21 | 0.29 | 0.46 |
| Mg | 0.2 | 0.24 | 0.21 |
| K | 1.4 | 1.89 | 1.52 |
| S | 0.1 | 0.16 | 0.11 |
| Cl | 0.25 | 1.08 | 0.32 |

DM, dry matter; RUP, rumen undegradable protein; CP, crude protein; NDF, neutral detergent fiber; ADF, acid detergent fiber; Ca, calcium; P, phosphide; Na, sodium; K, potassium; Mg, magnesium; NFC, Non Fiber Carbohydrates; S, Sulfur; Cl, Chlorine.
