# Supplementary material for: Effects of forage type on the rumen microbiota, growth performance, carcass traits, and meat quality in fattening goats
Source: Front Vet Sci. 2023 Apr 27;10:1147685. doi: 10.3389/fvets.2023.1147685 (PMC10172669; doi:10.3389/fvets.2023.1147685)
Supplement: Supplementary file 2 [file Table_2.DOCX]

Table S2: Amino acids (g/100g dry matter) and fatty acids (g/100g dry matter) of goat meat in different treatments.

| Traits^1^ | | Groups | | |
| --- | --- | --- | --- | --- |
|  |  | HA | PS | FG |
| *Longissimus dorsi* | Asn | 5.59±0.55 | 4.59.19±1.08 | 5.72±0.64 |
|  | Glu | 11.42±0.73 | 11.124±1.76 | 10.41±0.99 |
|  | Ser | 2.32±0.11^ABa^ | 2.56±0.40^Aa^ | 1.83±0.14^Bb^ |
|  | His | 3.51±0.17 | 3.18±0.60 | 318±0.57 |
|  | Gly | 3.71±0.35 | 4.33±1.02 | 3.34±0.54 |
|  | Thr | 2.98±1.28 | 3.02±0.48 | 2.57±0.23 |
|  | Arg | 5.32±0.34 | 5.41±0.97 | 4.82±0.45 |
|  | Ala | 4.09±0.20 | 4.14±0.78 | 3.70±0.37 |
|  | Tyr | 2.88±0.15 | 2.82±0.43 | 2.55±0.23 |
|  | Cys | 0.44±0.04 | 0.49±0.06 | 0.42±0.05 |
|  | Val | 4.96±0.24 | 4.76±0.77 | 4.58±0.49 |
|  | Met | 2.06±0.11 | 1.96±0.30 | 1.85±0.20 |
|  | Phe | 6.44±0.33 | 6.09±0.94 | 5.98±0.64 |
|  | Ile | 2.62±0.13 | 2.60±0.46 | 2.37±0.25 |
|  | Leu | 8.18±0.36 | 7.95±1.26 | 7.50±0.79 |
|  | Lys | 6.75±0.45 | 6.62±0.90 | 6.24±0.63 |
|  | Pro | 1.73±0.74^ab^ | 3.12±2.13^a^ | 0.91±0.76^b^ |
|  | C4:0 (butyric acid) | 0.00±0.00 | 0.00±0.00 | 0.00±0.00 |
|  | C6:0 (caproic acid) | 0.00±0.00 | 0.00±0.00 | 0.00±0.00 |
|  | C8:0 (caprylic acid) | 0.00±0.00 | 0.00±0.00 | 0.00±0.00 |
|  | C10:0 (capric acid) | 0.00±0.00 | 0.00±0.00 | 0.00±0.00 |
|  | C11:0 (undecanoic acid) | 0.00±0.00 | 0.00±0.00 | 0.00±0.00 |
|  | C12:0 (lauric acid) | 0.00±0.01 | 0.00±0.00 | 0.00±0.00 |
|  | C13:0 (ginkgolic acids) | 0.00±0.00 | 0.00±0.00 | 0.00±0.00 |
|  | C14:0 (myristic acid) | 0.11±0.06 | 0.07±0.04 | 0.10±0.05 |
|  | C14:1n5 (cis-myristoleic acid) | 0.00±0.01 | 0.00±0.00 | 0.00±0.00 |
|  | C15:0 (pentadecanoic acid) | 0.03±0.01 | 0.02±0.01 | 0.03±0.02 |
|  | C15:1n5 (methyl pentadecenoate) | 0.00±0.00 | 0.00±0.00 | 0.00±0.00 |
|  | C16:0 (palmitic acid) | 1.41±0.44 | 1.38±0.78 | 1.75±0.90 |
|  | C16:1n7 (palmitoleic acid) | 0.13±0.04 | 0.12±0.06 | 0.15±0.07 |
|  | C17:0 (heptadecanoic acid) | 0.08±0.02 | 0.09±0.06 | 0.10±0.06 |
|  | C17:1n7 (margaric acid) | 0.00±0.00 | 0.00±0.00 | 0.00±0.00 |
|  | C18:0 (stearic acid) | 1.08±0.04 | 1.26±0.79 | 1.69±1.06 |
|  | C18:1n9t (elaidic acid) | 0.00±0.00 | 0.00±0.00 | 0.01±0.01 |
|  | C18:1n9c (oleic acid) | 2.57±0.55 | 2.78±1.84 | 3.72±2.15 |
|  | C18:2n6t (linolelaidic acid) | 0.00±0.00 | 0.00±0.00 | 0.00±0.00 |
|  | C18:2n6c (linoleic acid) | 1.03±0.23 | 0.98±0.42 | 0.80±0.36 |
|  | C20:0 (behenic acid) | 0.00±0.00 | 0.00±0.00 | 0.00±0.00 |
|  | C18:3n6 (γ-linolenic acid) | 0.00±0.00 | 0.00±0.01 | 0.00±0.00 |
|  | C20:1 (eicosamonoenoic acid) | 0.00±0.00 | 0.01±0.01 | 0.00±0.01 |
|  | C18:3n3 (α-linolenic acid) | 0.03±0.01 | 0.03±0.01 | 0.02±0.01 |
|  | C21:0 (heneicosanoic acid) | 0.00±0.00 | 0.00±0.00 | 0.00±0.00 |
|  | C20:2 (eicosadienoic acid) | 0.00±0.00 | 0.00±0.01 | 0.00±0.00 |
|  | C22:0 (behenic acid) | 0.00±0.00 | 0.01±0.01 | 0.00±0.00 |
|  | C20:3n6 (dihomo-γ-linolenic acid) | 0.04±0.01 | 0.04±0.02 | 0.03±0.01 |
|  | C22:1n9 (erucic acid) | 0.00±0.00 | 0.01±0.01 | 0.00±0.01 |
|  | C20:3n3 (eicosatrienoic acid) | 0.00±0.00 | 0.00±0.00 | 0.00±0.00 |
|  | C20:4n6 (arachidonic acid) | 0.41±0.10 | 0.44±0.16 | 0.31±0.16 |
|  | C23:0 (tricosanoic acid) | 0.00±0.00 | 0.00±0.00 | 0.00±0.00 |
|  | C22:2n6 (docosadienoic acid) | 0.00±0.00 | 0.00±0.00 | 0.00±0.00 |
|  | C24:0 (tetracosanoic acid) | 0.00±0.00 | 0.00±0.00 | 0.00±0.00 |
|  | C20:5n3 (eicosapentaenoic acid) | 0.01±0.01 | 0.01±0.02 | 0.00±0.01 |
|  | C24:1n9 (nervonic acid) | 0.00±0.00 | 0.00±0.00 | 0.00±0.00 |
|  | C22:6n3 (docosahexaenoic acid) | 0.08±0.04 | 0.08±0.02 | 0.07±0.03 |
| *Semimembranosus* | Asn | 6.20±0.24^A^ | 3.86±0.11^B^ | 4.47±0.82^B^ |
|  | Glu | 11.77±0.30^a^ | 10.62±0.20 ^b^ | 11.33±0.87^b^ |
|  | Ser | 2.25±0.09 ^ABa^ | 2.64±0.06^Aa^ | 1.72±0.37^Bb^ |
|  | His | 2.96±0.13 | 2.84±0.19 | 2.84±0.26 |
|  | Gly | 4.44±0.48 | 3.92±0.52 | 4.35±0.33 |
|  | Thr | 2.90±0.10^A^ | 2.97±0.03^A^ | 2.52±0.34^B^ |
|  | Arg | 5.49±0.09 | 5.03±0.14 | 5.22±0.42 |
|  | Ala | 4.29±0.16^a^ | 3.84±0.14^b^ | 4.11±0.26^ab^ |
|  | Tyr | 2.83±0.11 | 2.71±0.03 | 2.57±0.28 |
|  | Cys | 0.44±0.04^B^ | 0.50±0.02^A^ | 0.40±0.02^B^ |
|  | Val | 4.92±0.17^A^ | 4.22±0.03^B^ | 4.78±0.37^A^ |
|  | Met | 2.01±0.08 | 1.88±0.03 | 1.92±0.17 |
|  | Phe | 6.33±0.26^A^ | 5.36±0.06^B^ | 6.14±0.51^A^ |
|  | Ile | 2.65±0.08 | 2.46±0.05 | 2.55±0.21 |
|  | Leu | 8.12±0.30 | 7.47±0.05 | 7.85±0.64 |
|  | Lys | 6.80±0.20 | 6.73±0.26 | 6.84±0.53 |
|  | Pro | 1.83±0.62^b^ | 4.02±1.15^ab^ | 4.72±2.05^a^ |
|  | C4:0 (butyric acid) | 0.00±0.00 | 0.00±0.00 | 0.00±0.00 |
|  | C6:0 (caproic acid) | 0.00±0.00 | 0.00±0.00 | 0.00±0.00 |
|  | C8:0 (caprylic acid) | 0.00±0.00 | 0.00±0.00 | 0.00±0.00 |
|  | C10:0 (capric acid) | 0.00±0.00 | 0.00±0.00 | 0.00±0.00 |
|  | C11:0 (undecanoic acid) | 0.00±0.00 | 0.00±0.00 | 0.00±0.00 |
|  | C12:0 (lauric acid) | 0.00±0.00 | 0.00±0.00 | 0.00±0.00 |
|  | C13:0 (ginkgolic acids) | 0.00±0.00 | 0.00±0.00 | 0.00±0.00 |
|  | C14:0 (myristic acid) | 0.08±0.02 | 0.06±0.01 | 0.05±0.04 |
|  | C14:1n5 (cis-myristoleic acid) | 0.00±0.00 | 0.00±0.00 | 0.00±0.00 |
|  | C15:0 (pentadecanoic acid) | 0.01±0.01 | 0.01±0.01 | 0.01±0.01 |
|  | C15:1n5 (methyl pentadecenoate) | 0.00±0.00 | 0.00±0.00 | 0.00±0.00 |
|  | C16:0 (palmitic acid) | 0.82±0.19 | 0.73±0.21 | 1.03±0.57 |
|  | C16:1n7 (palmitoleic acid) | 0.08±0.03 | 0.07±0.01 | 0.10±0.06 |
|  | C17:0 (heptadecanoic acid) | 0.04±0.02 | 0.04±0.02 | 0.05±0.03 |
|  | C17:1n7 (margaric acid) | 0.00±0.00 | 0.00±0.00 | 0.00±0.00 |
|  | C18:0 (stearic acid) | 0.71±0.36 | 0.60±0.22 | 0.97±0.58 |
|  | C18:1n9t (elaidic acid) | 0.00±0.00 | 0.00±0.00 | 0.02±0.05 |
|  | C18:1n9c (oleic acid) | 1.51±0.67 | 1.26±0.44 | 2.19±1.29 |
|  | C18:2n6t (linolelaidic acid) | 0.00±0.00 | 0.00±0.00 | 0.00±0.00 |
|  | C18:2n6c (linoleic acid) | 0.76±0.35 | 0.51±0.20 | 0.97±0.38 |
|  | C20:0 (behenic acid) | 0.00±0.00 | 0.01±0.01 | 0.00±0.00 |
|  | C18:3n6 (γ-linolenic acid) | 0.00±0.00 | 0.00±0.00 | 0.00±0.00 |
|  | C20:1 (eicosamonoenoic acid) | 0.00±0.00 | 0.00±0.00 | 0.00±0.01 |
|  | C18:3n3 (α-linolenic acid) | 0.02±0.01 | 0.01±0.01 | 0.02±0.01 |
|  | C21:0 (heneicosanoic acid) | 0.00±0.00 | 0.00±0.00 | 0.00±0.00 |
|  | C20:2 (eicosadienoic acid) | 0.00±0.00 | 0.00±0.00 | 0.01±0.01 |
|  | C22:0 (behenic acid) | 0.00±0.00 | 0.00±0.00 | 0.00±0.00 |
|  | C20:3n6 (dihomo-γ-linolenic acid) | 0.03±0.01 | 0.02±0.02 | 0.04±0.00 |
|  | C22:1n9 (erucic acid) | 0.00±0.00 | 0.00±0.00 | 0.01±0.01 |
|  | C20:3n3 (eicosatrienoic acid) | 0.00±0.00 | 0.00±0.00 | 0.02±0.04 |
|  | C20:4n6 (arachidonic acid) | 0.30±0.19 | 0.22±0.12 | 0.47±0.12 |
|  | C23:0 (tricosanoic acid) | 0.00±0.00 | 0.00±0.00 | 0.00±0.00 |
|  | C22:2n6 (docosadienoic acid) | 0.00±0.00 | 0.00±0.00 | 0.00±0.00 |
|  | C24:0 (tetracosanoic acid) | 0.00±0.00 | 0.00±0.01 | 0.00±0.00 |
|  | C20:5n3 (eicosapentaenoic acid) | 0.01±0.01 | 0.01±0.01 | 0.00±0.00 |
|  | C24:1n9 (nervonic acid) | 0.00±0.00 | 0.00±0.00 | 0.00±0.00 |
|  | C22:6n3 (docosahexaenoic acid) | 0.06±0.03 | 0.04±0.03 | 0.09±0.02 |

The results are presented as means and standard errors.
