# Supplementary material for: Effects of forage type on the rumen microbiota, growth performance, carcass traits, and meat quality in fattening goats
Source: Front Vet Sci. 2023 Apr 27;10:1147685. doi: 10.3389/fvets.2023.1147685 (PMC10172669; doi:10.3389/fvets.2023.1147685)
Supplement: Supplementary file 3 [file Data_Sheet_1.PDF]

Bray Curtis group significance

Overview

A

	PERMANOVA results
**method name**	PERMANOVA
**test statistic name**	pseudo-F
**sample size**	13
**number of groups**	3
**test statistic**	0.994274
**p-value**	0.434
**number of permutations**	999

Group significance plots

Download raw data as TSV (raw_data.tsv)

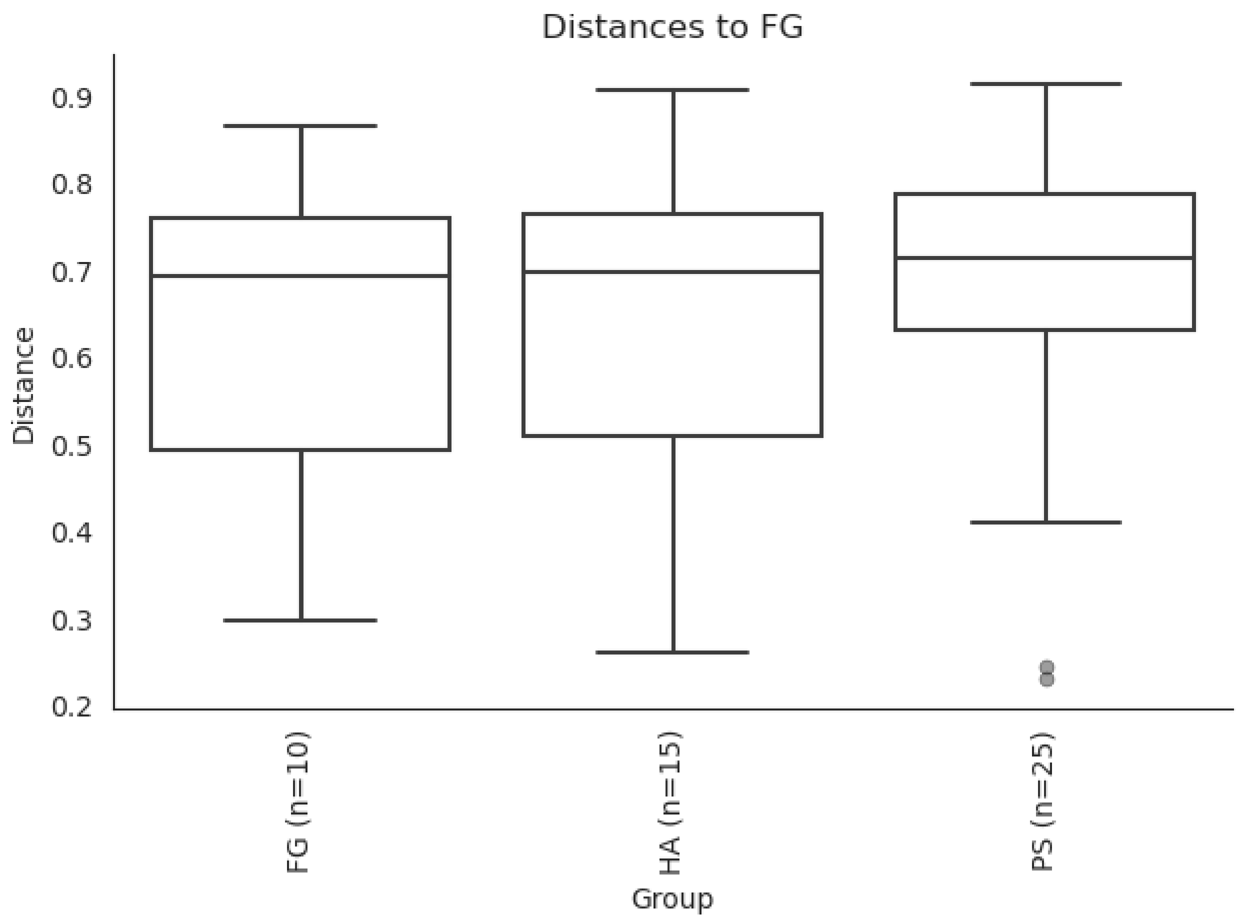

Download as PDF

(FG-boxplots.pdf)

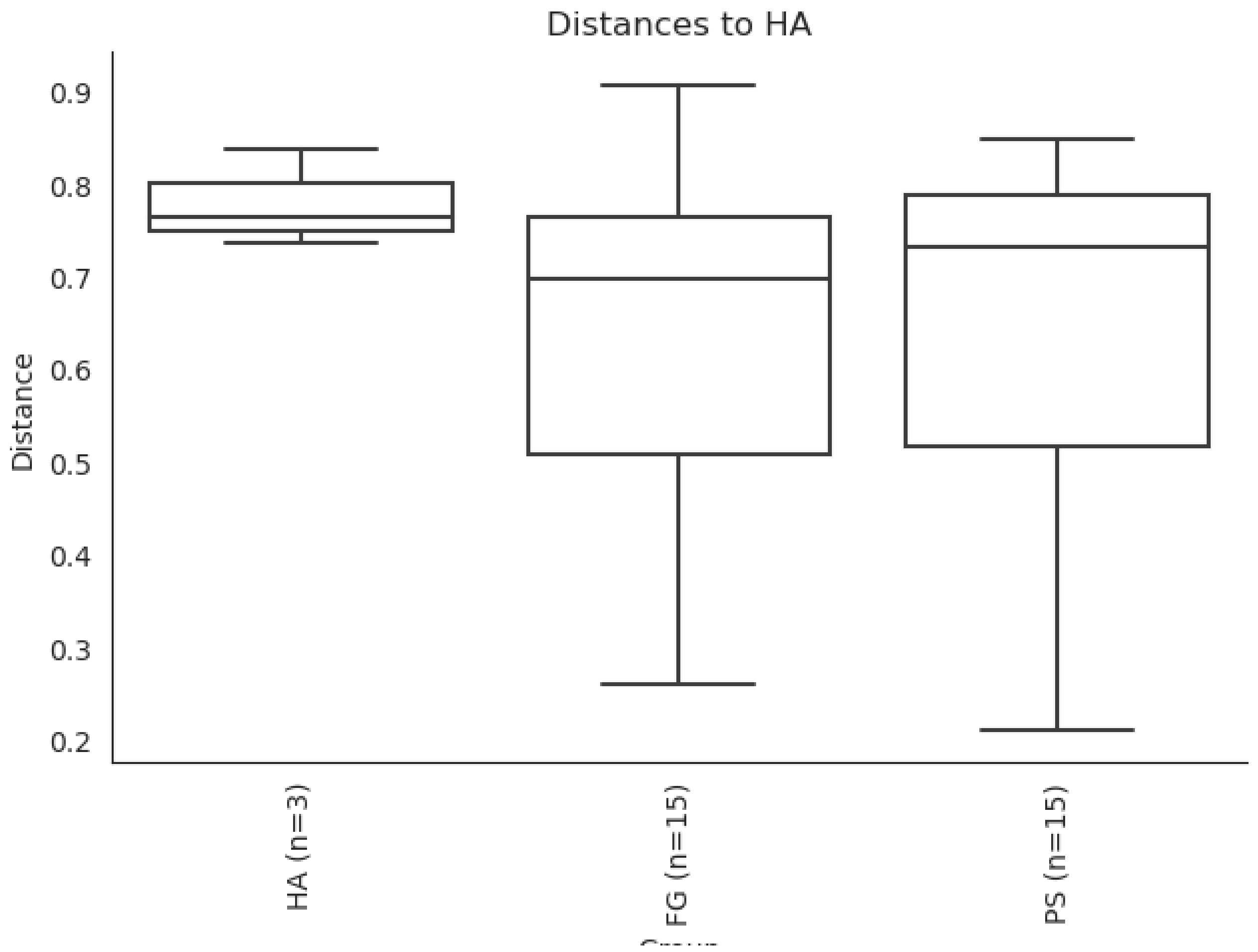

Download as PDF

(HA-boxplots.pdf)

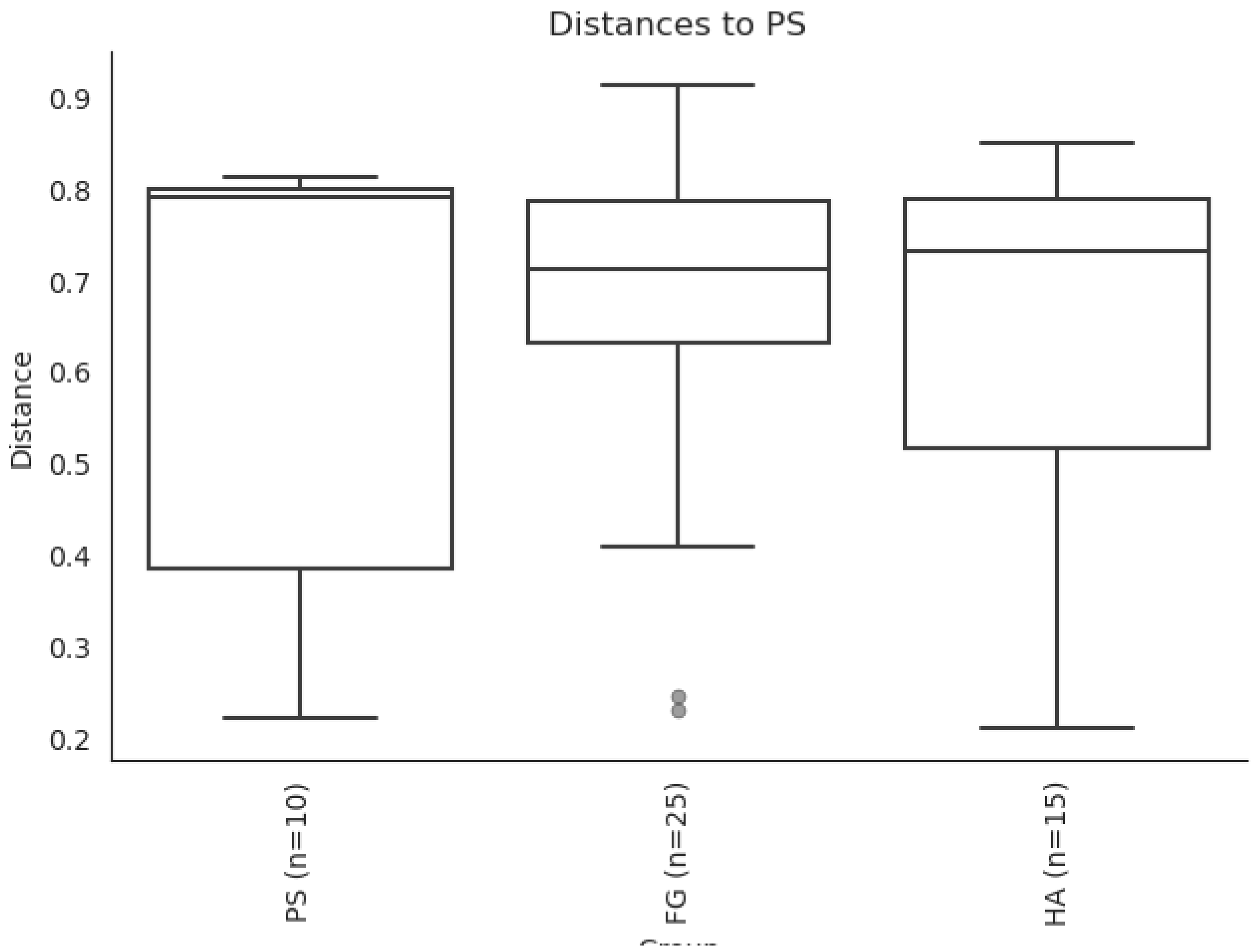

Download as PDF

(PS-boxplots.pdf)

Pairwise permanova results

Download CSV (permanova-pairwise.csv)

		Sample size	Permutations	pseudo-F	p-value	q-value
**Group 1**	**Group 2**					
**FG**	**HA**	8	999	0.456349	0.872	0.872
	**PS**	10	999	1.892987	0.092	0.276
**HA**	**PS**	8	999	0.543466	0.615	0.872

Overview

B

Jaccard Distance group significance

	PERMANOVA results
**method name**	PERMANOVA
**test statistic name**	pseudo-F
**sample size**	13
**number of groups**	3
**test statistic**	1.05539
**p-value**	0.38
**number of permutations**	999

Group significance plots

Download raw data as TSV (raw_data.tsv)

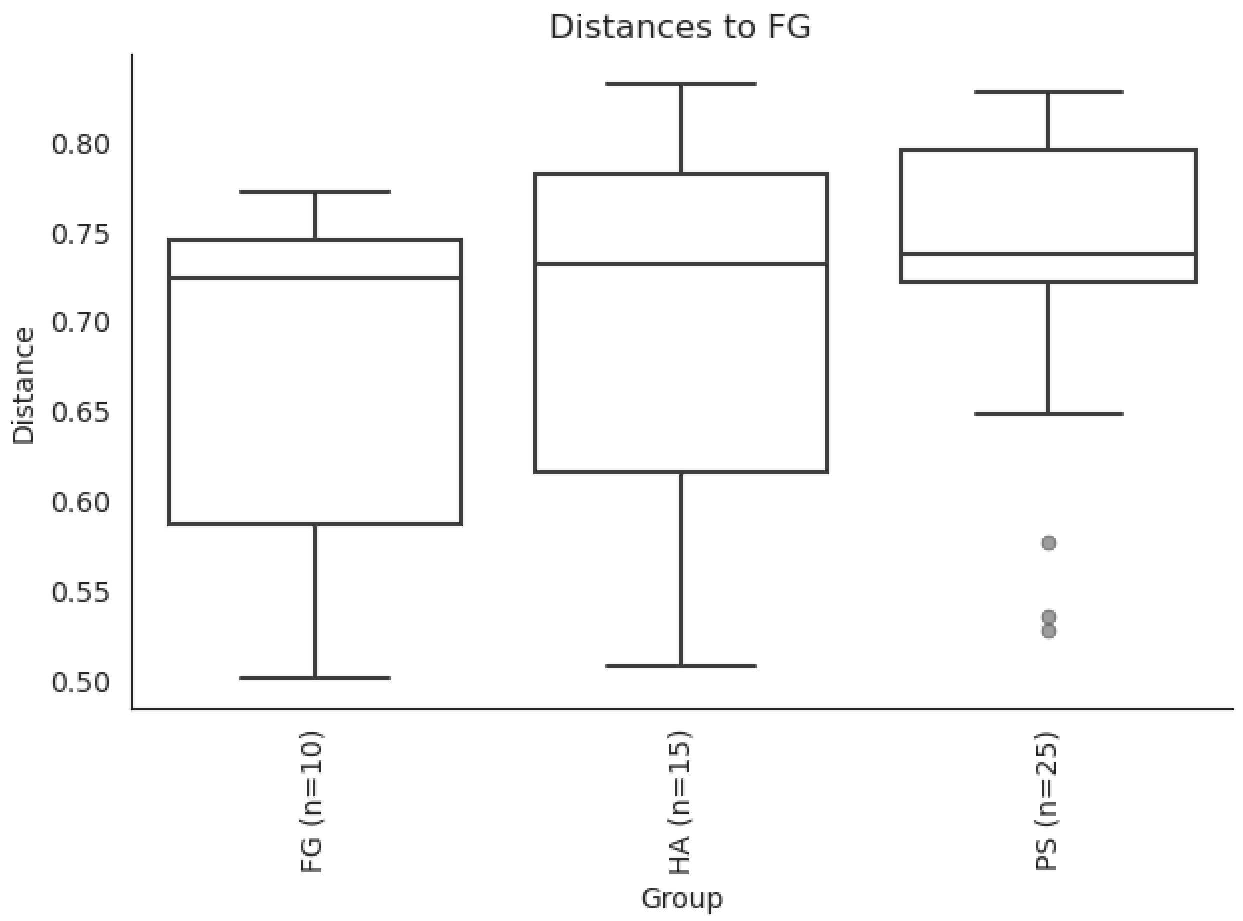

Download as PDF

(FG-boxplots.pdf)

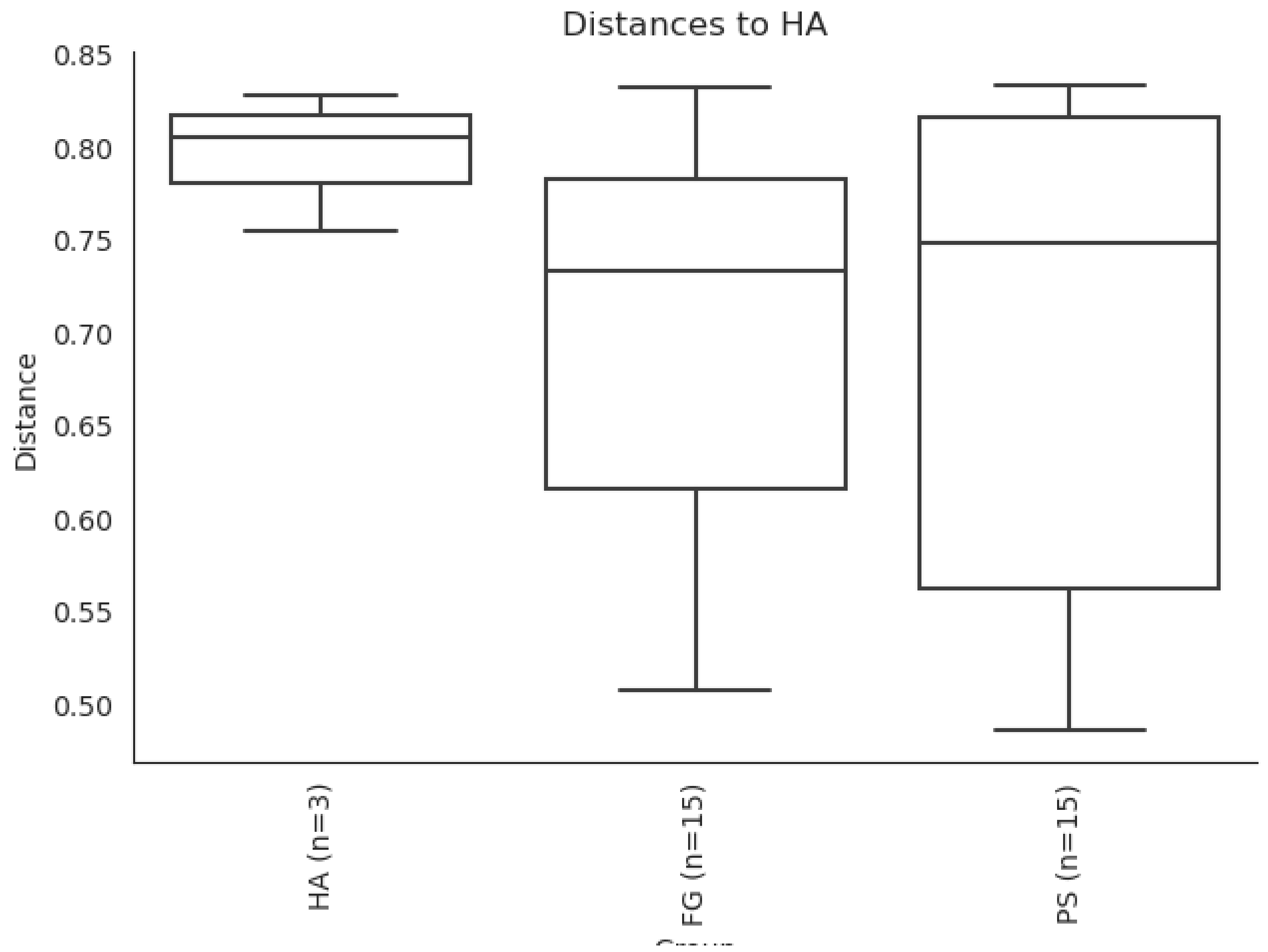

Download as PDF

(HA-boxplots.pdf)

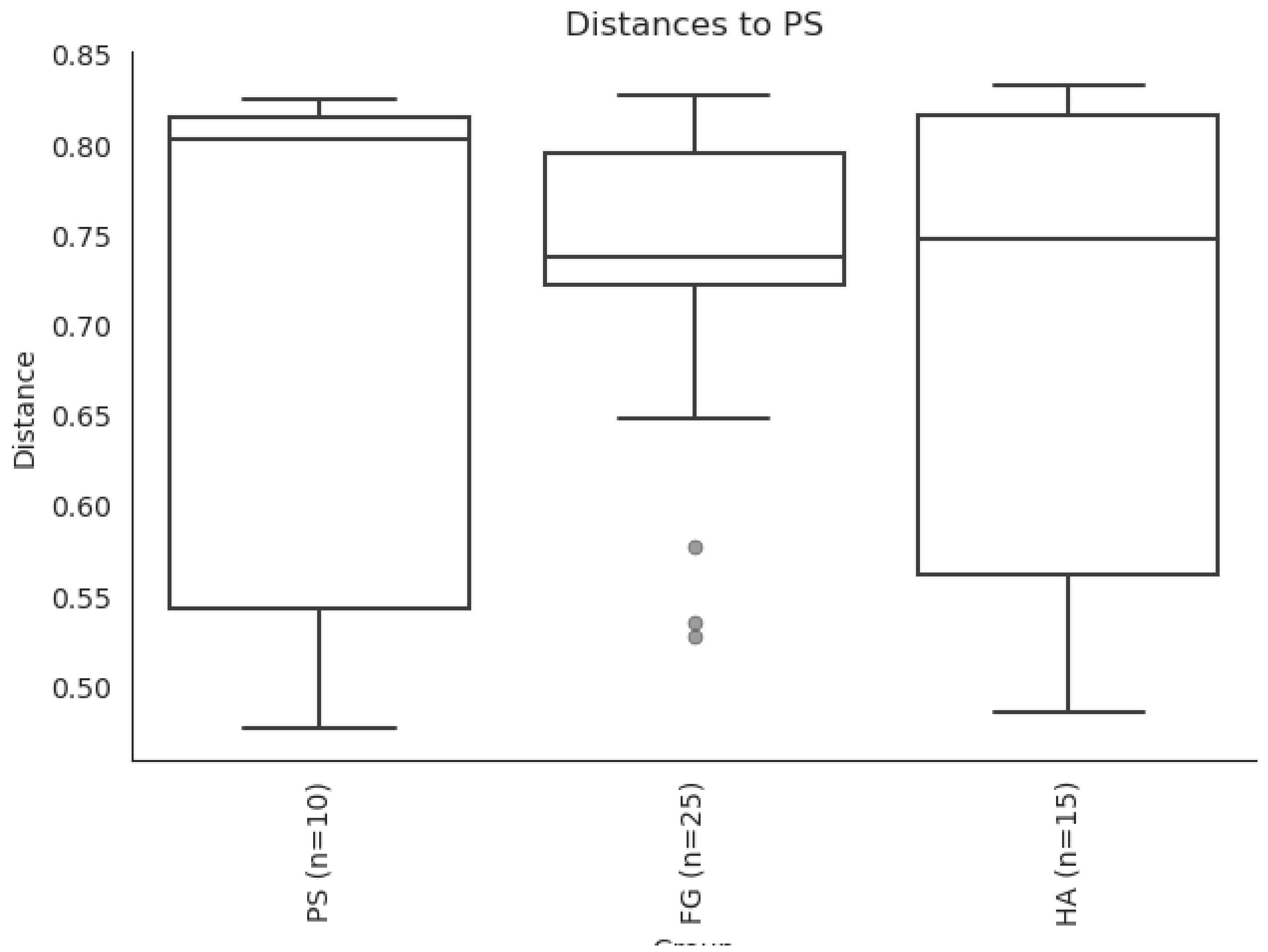

Download as PDF

(PS-boxplots.pdf)

Pairwise permanova results

Download CSV (permanova-pairwise.csv)

Group 1	Group 2	Sample size	Permutations	pseudo-F	p-value	q-value
**FG**	**HA**	8	999	0.764680	0.820	0.8200
	**PS**	10	999	1.613813	0.083	0.2490
**HA**	**PS**	8	999	0.710283	0.503	0.7545

Unweighted Unifrac group significance

Overview

C

	PERMANOVA results
**method name**	PERMANOVA
**test statistic name**	pseudo-F
**sample size**	13
**number of groups**	3
**test statistic**	1.24253
**p-value**	0.264
**number of permutations**	999

Group significance plots

Download raw data as TSV (raw_data.tsv)

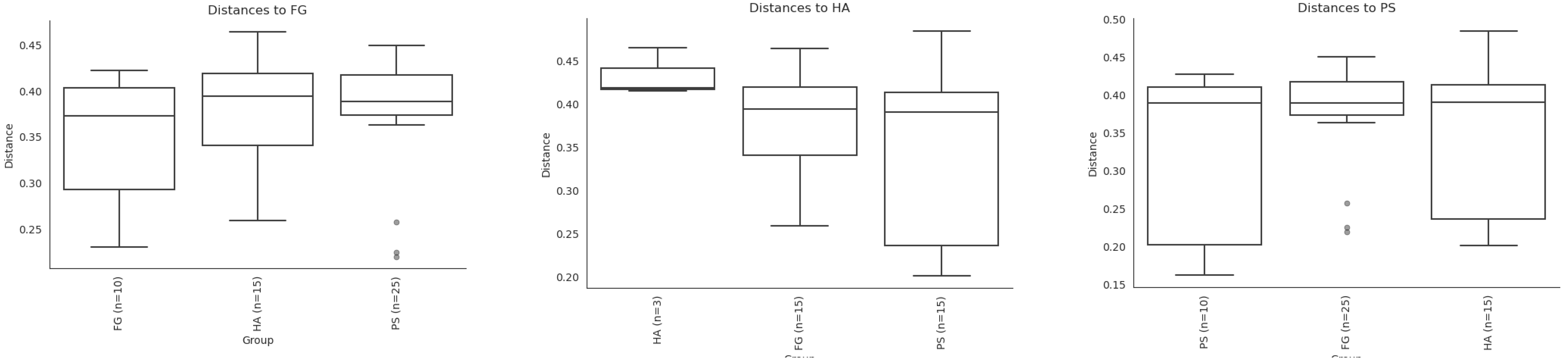

Download as PDF (FG-boxplots.pdf)

Download as PDF (HA-boxplots.pdf)

Download as PDF (PS-boxplots.pdf)

Pairwise permanova results

Download CSV (permanova-pairwise.csv)

Group 1	Group 2	Sample size	Permutations	pseudo-F	p-value	q-value
**FG**	**HA**	8	999	0.810576	0.652	0.652
	**PS**	10	999	2.147720	0.056	0.168
**HA**	**PS**	8	999	0.705579	0.512	0.652

Overview

D

Weighted Unifrac group significance

	PERMANOVA results
**method name**	PERMANOVA
**test statistic name**	pseudo-F
**sample size**	13
**number of groups**	3
**test statistic**	0.985364
**p-value**	0.398
**number of permutations**	999

Group significance plots

Download raw data as TSV (raw_data.tsv)

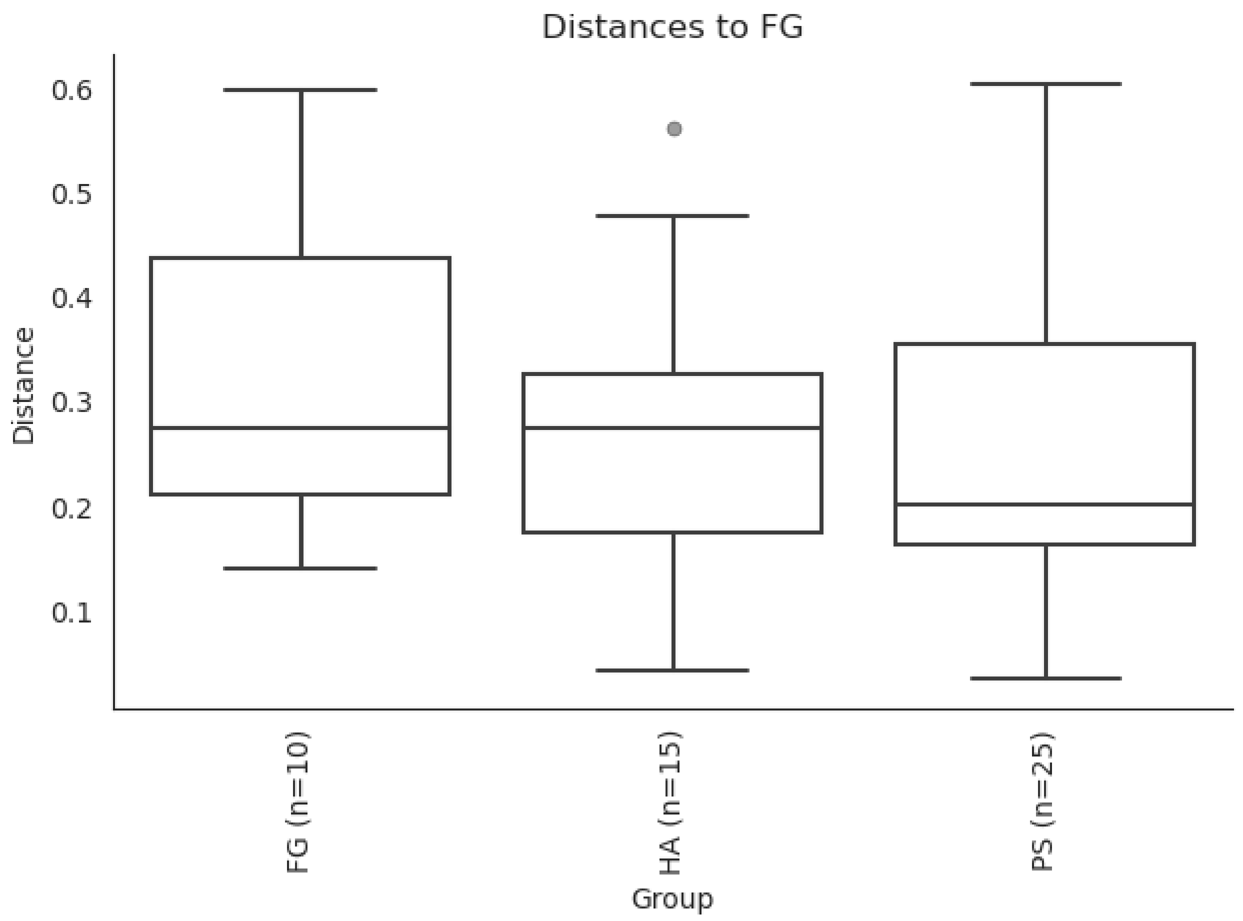

Download as PDF

(FG-boxplots.pdf)

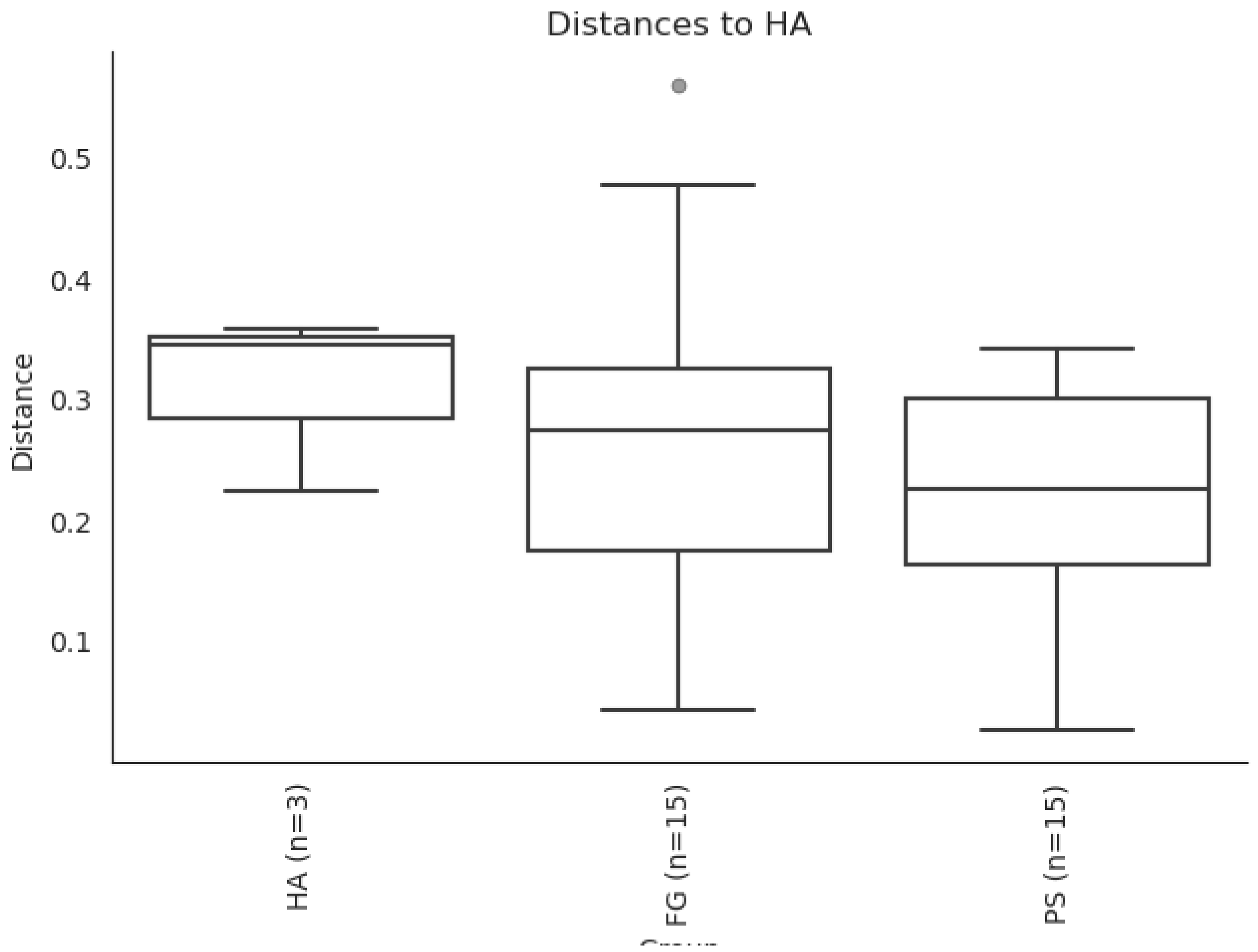

Download as PDF

(HA-boxplots.pdf)

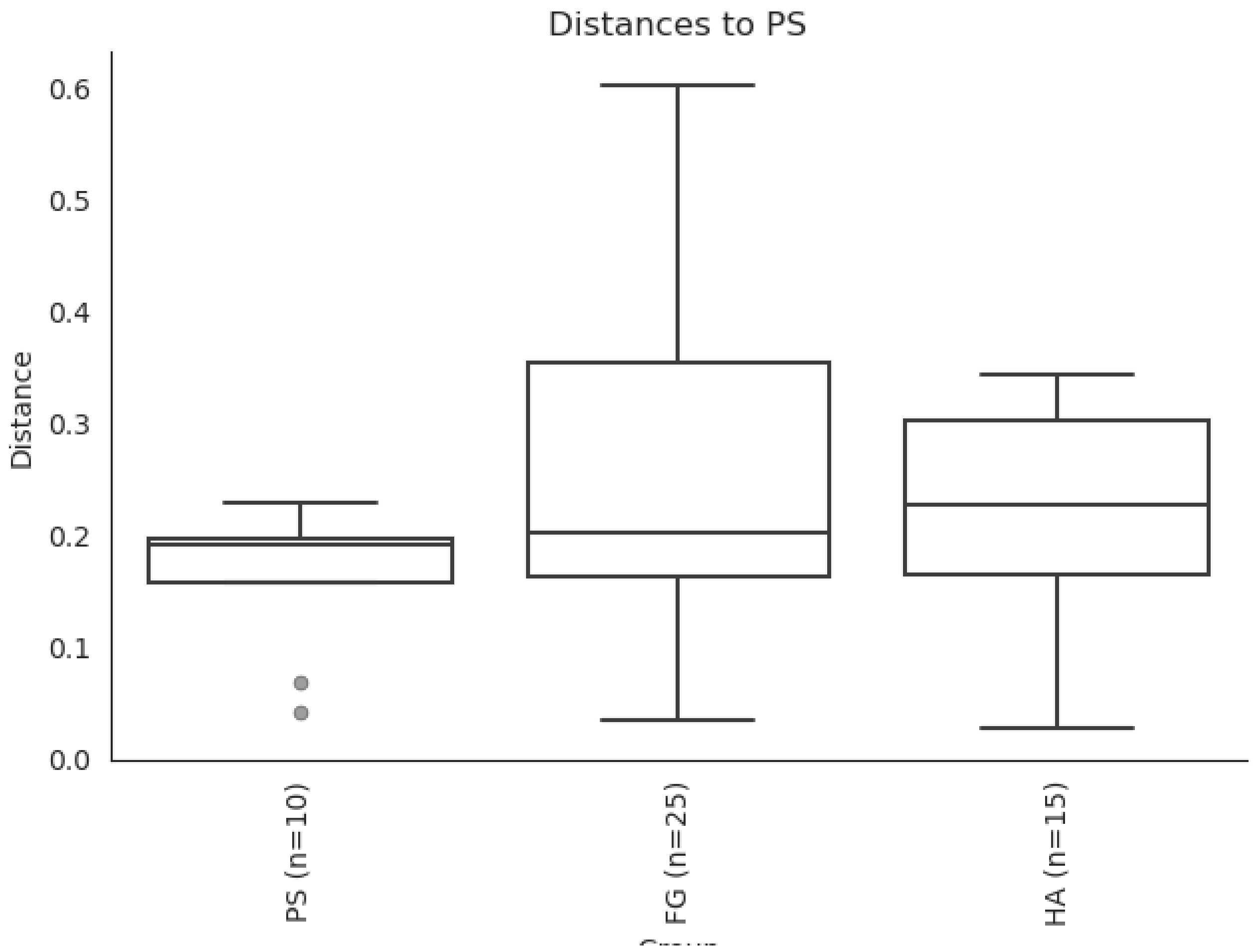

Download as PDF

(PS-boxplots.pdf)

Pairwise permanova results

Download CSV (permanova-pairwise.csv)

Group 1	Group 2	Sample size	Permutations	pseudo-F	p-value	q-value
**FG**	**HA**	8	999	0.142368	0.948	0.948
	**PS**	10	999	2.036457	0.160	0.480
**HA**	**PS**	8	999	0.904949	0.480	0.720
